# Draft genome sequences of two opportunistic pathogenic strains of *Staphylococcus cohnii* isolated from human patients

**DOI:** 10.1186/s40793-017-0263-1

**Published:** 2017-08-31

**Authors:** Soraya Mendoza-Olazarán, José F. Garcia-Mazcorro, Rayo Morfín-Otero, Licet Villarreal-Treviño, Adrián Camacho-Ortiz, Eduardo Rodríguez-Noriega, Paola Bocanegra-Ibarias, Héctor J. Maldonado-Garza, Scot E. Dowd, Elvira Garza-González

**Affiliations:** 1 Servicio de Gastroenterología, Hospital Universitario Dr. José Eleuterio González, Universidad Autónoma de Nuevo León, Monterrey, Nuevo León Mexico; 2 Facultad de Medicina Veterinaria, Universidad Autónoma de Nuevo León, General Escobedo, Nuevo León Mexico; 3 Hospital Civil de Guadalajara, Fray Antonio Alcalde, and Instituto de Patología Infecciosa y Experimental, Centro Universitario de Ciencias de la Salud, Universidad de Guadalajara, Guadalajara, Jalisco Mexico; 4 Departamento de Microbiología e Inmunología, Facultad de Ciencias Biológicas, Universidad Autónoma de Nuevo León, Monterrey, Nuevo León Mexico; 5 Servicio de Infectología, Hospital Universitario Dr. José Eleuterio González, Universidad Autónoma de Nuevo León, Monterrey, Nuevo León Mexico; 6 Molecular Research DNA Laboratory, Shallowater, TX USA

**Keywords:** Short genome report, *Staphylococcus cohnii*, Coagulase-negative staphylococci, Clinical strains, Biofilm, SCC*mec*

## Abstract

Herein, we report the draft-genome sequences and annotation of two opportunistic pathogenic strains of *Staphylococcus cohnii* isolated from humans. One strain (SC-57) was isolated from blood from a male patient in May 2006 and the other (SC-532) from a catheter from a male patient in June 2006.

Similar to other genomes of *Staphylococcus* species, most genes (42%) of both strains are involved in metabolism of amino acids and derivatives, carbohydrates and proteins. Eighty (4%) genes are involved in virulence, disease, and defense and both species show phenotypic low biofilm production and evidence of increased antibiotic resistance associated to biofilm production. From both isolates, a new Staphylococcal Cassette Chromosome *mec* was detected: *mec* class A, *ccr* type 1. This is the first report of whole genome sequences of opportunistic *S. cohnii* isolated from human patients.

**Electronic supplementary material:**

The online version of this article (10.1186/s40793-017-0263-1) contains supplementary material, which is available to authorized users.

## Introduction

CoNS are opportunistic pathogens in humans and other animal species. Some of these species are normal microbiota of human skin and mucous membranes and are frequently detected as contaminants of microbiological cultures from clinical specimens [1, 2]. The increasing frequency of CoNS as opportunistic pathogens has been attributed in part to the use of medical devices, such as intravascular catheters and prostheses [3]. The increase has been related to the production of biofilm by some CoNS species since biofilm allows the adherence of bacteria to plastic medical devices. The biofilm may protect bacteria against immunological host defense mechanisms and antimicrobial therapy [4]. The biofilm is composed of polysaccharides, proteins and DNA. In 10.1601/nm.5246 the biofilm formation has been associated mainly with the production of PIA encoded by the *ica* operon [5].

10.1601/nm.11046 belong of the CoNS group and has been isolated from human and non-human primates [6]. This species is an important opportunistic pathogen for humans, which has been associated to blood stream infection, endocarditis and meningitis [7, 8]. There is only one published draft genome sequence of one strain of 10.1601/nm.11046 isolated from a hospital environment in China, but none has been sequenced from human sources [9]. Here, we report the draft-genome sequences and annotation of two opportunistic strains of 10.1601/nm.11046 isolated from human patients. One strain was isolated from blood in May 2006 and the other strain from a catheter in June 2006.

## Organism information

### Classification and features

**Fig. 1:**
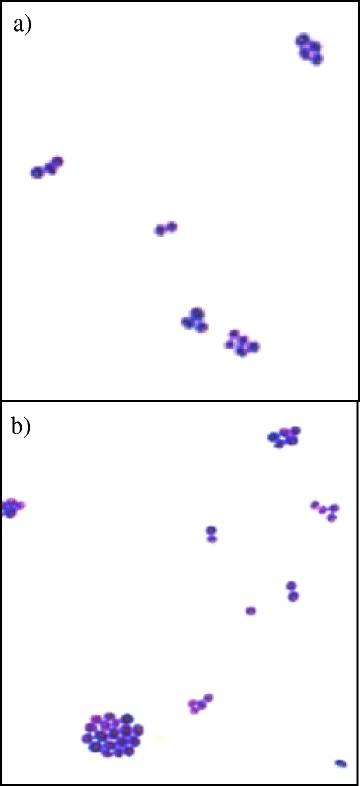
Gram stain of isolates SC-57 (**a**) and SC -532 (**b**) using light microscopy at magnification 100×

10.1601/nm.11046 strains SC-57 and SC-532 were classified as causative agents of bacteremia and catheter-related blood stream infection, respectively. Strains were recovered from patients in a tertiary hospital in Monterrey, Mexico. For light microscopy, cells were observed with a Zeiss Axio Imager A1 (Jena, Germany) microscope. Cells were stained as Gram-positive and presented a spherical shape in the exponential growth phase (Fig. 1). Classification and general features of isolates SC-57 and SC-532 in accordance with MIGS specifications [10] are shown in Table 1.

**Table 1:** Classification and general features of *Staphylococcus cohnii* strains SC-57 and SC-532 [10]

MIGS ID	Property	Term	Evidence code^a^
	Classification	Domain *Bacteria*	TAS [44]
		Phylum *Firmicutes*	TAS [45]
		Class *Bacilli*	TAS [46]
		Order *Bacillales*	TAS [47]
		Family *Staphylococcaceae*	TAS [48]
		Genus *Staphylococcus*	TAS [49]
		Species *Staphylococcus cohnii*	TAS [11]
		Strains: SC-57 and SC-532	IDA
	Gram stain	Positive	IDA
	Cell shape	coccus	IDA
	Motility	Nonmotile	IDA
	Sporulation	Nonsporulating	IDA
	Temperature range	15–45 °C	IDA
	Optimum temperature	37 °C	IDA
	pH range; Optimum	6.5–7.5, 7	IDA
	Carbon source	D-mannitol, fructose, trehalose, glucose, mannose, lactose,	IDA
MIGS-6	Habitat	Skin	IDA
MIGS-6.3	Salinity	Tolerates 10% NaCl (*w*/*v*)	IDA
MIGS-22	Oxygen requirement	Facultative anaerobic	IDA
MIGS-15	Biotic relationship	Free living	IDA
MIGS-14	Pathogenicity	Opportunistic pathogenic	IDA
MIGS-4	Geographic location	Monterrey, Mexico	IDA
MIGS-5	Sample collection	May 23, 2006 (SC-57), June 8, 2006 (SC-532)	IDA
MIGS-4.1	Latitude	25.6714.	IDA
MIGS-4.2	Longitude	−100.309	IDA
MIGS-4.4	Altitude	534 m	IDA

^a^ Evidence codes - IDA: Inferred from Direct Assay; TAS: Traceable Author Statement (i.e., a direct report exists in the literature); NAS: Non-traceable Author Statement (i.e., not directly observed for the living, isolated sample, but based on a generally accepted property for the species, or anecdotal evidence). These evidence codes are from the Gene Ontology project [19]

**Fig. 2:**
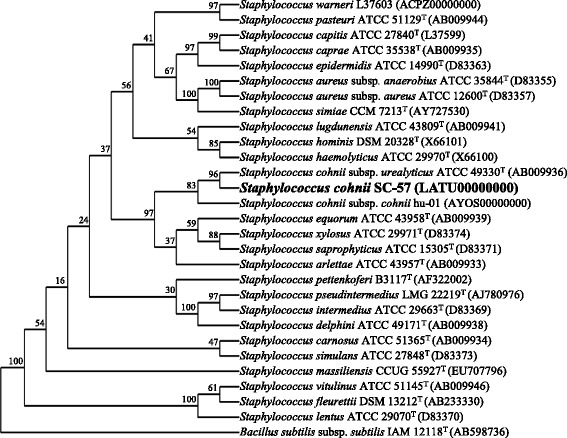
Phylogenetic tree based on 16S rRNA gene sequences of the genus *Staphylococcus*. The names and corresponding accession numbers are shown, including the *S. cohnii* (SC-57) sequence from this study, which was 100% similar to SC-532. Sequences were aligned using Clustal W2 and MUSCLE [13] and uploaded in DAMBE [15] to build a phylogenetic tree using a Maximum Likelihood method with the GTR substitution model, rate heterogeneity among sites modeled by a gamma distribution, and 1000 bootstrap samples. The number at the nodes represents bootstrap support. Generated with the ‘Quick add’ option on, and the number of branches allowed to cross during tree searching set to 1 for local optimization. *Bacillus subtilis subsp. subtilis* (AB598736) was chosen as the out-group to root the tree

16S rRNA partial sequence of 10.1601/nm.11046 strain 10.1601/strainfinder?urlappend=%3Fid%3DATCC+49330 (AB009936) showed identity of 100% with the strains of this study. All 16S rRNA sequences found in our strains were 100% similar; therefore, we only used one sequence for phylogenetic analysis. Figure 2 shows a phylogenetic tree of the 16S rRNA gene of our representative strain (SC-57) and selected 16S rRNA sequences of the others 10.1601/nm.5230 species [9]. To building meaningful phylogenetic trees, we choose the FindModel tool available at the HIV Molecular Immunology Database because it allow a correct model nucleotide substitution [11] (GTR or GTR plus Gamma was selected based on the Akaike information criterion, initial tree constructed using Weighbor [12]). Sequences were aligned using Clustal W2 and MUSCLE [13, 14], and uploaded in DAMBE [15] to build a phylogenetic tree using a Maximum Likelihood method. Our results indicate an identical tree topology compared to the one in Hu et al. [9], with our sequence being more closely related to 10.1601/nm.5243 [16] (Fig. 2).

#### Extended feature descriptions

Both strains were identified as CoNS based on colony morphology, Gram staining, catalase test (positive), and coagulase test (negative). The strains were identified to the species level using the API Staph kit (bioMérieux, France), which consists of plastic microtubes containing 20 tests with dehydrated substrates to detect the enzymatic activity or the assimilation / fermentation of sugars by the inoculated organisms. On API system, both isolates were positive for medium acidification due to fermentation of glucose, fructose, mannose, maltose, lactose, trehalose and *N*-acetyl-glucosamine, production of *N*-acetyl-methyl-carbinol (Voges-Proskauer) and urease. Isolates were negative for fermentation of xylitol, melibiose, raffinose, xylose, saccharose, methyl-αD-glucopyranoside, reduction of nitrates to nitrites and arginine dihydrolase. The identification was confirmed by the MALDI-TOF system. 10.1601/nm.11046 10.1601/strainfinder?urlappend=%3Fid%3DATCC+49330 was used as control organism.

## Genome sequencing information

### Genome project history

The two genomes were selected for sequencing on the basis of their clinical relevance and isolation source. Sequencing and annotation were performed at the Molecular Research DNA Laboratory, Shallowater, Texas, United States of America. The draft genomes sequences were obtained on November 21, 2014.

The genome projects are deposited in the Genomes OnLine Database under accession numbers Gp0119449 (SC-57) and Gp0119450 (SC-532). This Whole Genome Shotgun project has been deposited at DDBJ/EMBL/GenBank under the accessions NZ_LATU00000000 (SC-57) and NZ_LATV00000000 (SC-532). The versions described in this paper are versions NZ_LATU00000000.1 and NZ_LATV00000000.1, respectively. The project information and its association with MIGS version 2.0 compliance are presented in Table 2 [10].

**Table 2:** Project information

MIGS ID	Property	SC-57	SC-532
MIGS-31	Finishing quality	High-quality draft	High-quality draft
MIGS-28	Libraries used	2 × 250 bp	2 × 250 bp
MIGS-29	Sequencing platforms	MiSeq Illumina	MiSeq Illumina
MIGS-31.2	Fold coverage	>40× (based on 500 bp library)	>40× (based on 500 bp library)
MIGS-30	Assemblers	NGEN-Assembler	NGEN-Assembler
MIGS-32	Gene calling method	NCBI PGAP pipeline	NCBI PGAP pipeline
	Locus Tag	XA22	XA21
	GenBank ID	NZ_LATU00000000	NZ_LATV00000000
	GenBank date of Release	April 15, 2015	April 15, 2015
	GOLD ID	Gp0119449	Gp0119450
	BIOPROJECT	PRJNA279286	PRJNA279286
MIGS-13	Source Material Identifier	SAMN03449103	SAMN03449104
	Project relevance	Clinical	Clinical

### Growth conditions and genomic DNA preparation

For isolate SC-57 a blood culture bottle was incubated using the Versatrek system (TREK Diagnostic Systems, Oakwood Village, Ohio). Subculture of the bottle was performed on 5% blood agar, and the plate was incubated at 35 °C for 24–48 h. The SC-532 isolate was cultured from a catheter tip using the Maki method [17]. After biochemical identification, species was confirmed by partial sequencing of the 16S rRNA gene [18]. Sequencing was performed at the Instituto de Biotecnología, Universidad Nacional Autónoma de México. DNA sequences were compared to genes in the National Center for Biotechnology Information GenBank using the BLAST algorithm [19]. For genome sequencing, genomic DNA was obtained using a commercial DNA extraction kit (QIAamp DNA Mini Kit, CA, USA). The concentration and purity of DNA was measured in a Spectrophotometer Beckman DU 640 (Minnesota, USA). Pure DNA was sent to Molecular Research LP (Shallowater, TX, USA).

### Genome sequencing and assembly

Deep sequencing was carried out using Illumina MiSeq. DNA libraries were prepared using Nextera DNA sample prep kits to create individual barcode indices. At least 0.8 gigabases of nucleotide sequences were generated. The assembly was performed by method NGEN v12 default paired end sequencing parameters (2 × 250 bp sequencing). The genome coverage was 40.0× with >1 million reads. The number of contigs in SC-57 and SC-532 were 20 and 16, respectively. Average size of contigs was 142,672 bp (SC-57) and 114,467 bp (SC-532).

### Genome annotation

The generated assembled and unassembled data were analyzed using MG-RAST metagenome analysis server [20]. An evidence-based annotation approach was used for annotation of metagenomic sequences [20, 21]. Sequences were analyzed using BlastX against protein databases with an E-value cutoff of 1 × 10^−5^. Predicted genes were classified and tabulated into functional categories from lower (individual genes) to higher (cellular processes) orders. The draft genomes were annotated using the standard operation procedure of the GenBank and IMG Expert Review platform developed by the Joint Genome Institute, Walnut Creek, CA, USA under IMG genome ID 2623620626 (SC-57) and 2651869670 (SC-532) [22]. For the prediction of signal peptides and transmembrane domains, SignalP 4.1 Server [23, 24] and TMHMM Server v. 2.0 [25] were used, respectively. Assignment of genes to the COG database [26, 27] and Pfam domains [28] were performed with WebMGA server [29]. CRISPR regions were identified with CRISPRFinder [30, 31].

## Genome properties

The total genome of SC-57 was 2,853,167 bp in size. The reads were assembled into 20 contigs with 80 RNAs (18 rRNA, 58 tRNA and 4 ncRNA) and 2699 CDSs. The total genome of SC-532 was 2,826,849 bp in size. The reads were assembled into 16 contigs with 78 RNAs (17 rRNA, 57 tRNA and 4 ncRNA) and 2677 CDSs (Table 3). The distribution of genes into COG functional categories is presented in Table 4. Similar to other genomes, most genes (42%) of both strains are involved in metabolism of amino acids and derivatives, carbohydrates, and proteins [32]. Eighty genes (4%) are involved in virulence, disease, and defense.

**Table 3:** Genome statistics

	SC-57		SC-532	
Attribute	Value	% of Total	Value	% of Total
Genome size (bp)	2,853,167	100.00	2,826,849	100.00
DNA coding (bp)	2,852,026	99.96	2,404,235	85.05
DNA G + C (bp)	951,817	33.36	943,037	33.36
DNA scaffolds	20	100.00	16	100.00
Total genes	2779	100.00	2755	100.00
Protein coding genes	2635	100.00	2620	100.00
RNA genes	80	3.04	78	2.98
Pseudo genes	64	2.32	57	2.15
Genes in internal clusters	232	8.75	211	7.99
Genes with function prediction	2256	85.13	2155	81.63
Genes assigned to COGs	1999	75.43	1982	75.08
Genes with Pfam domains	2284	86.19	2263	85.72
Genes with signal peptides	61	2.30	61	2.31
Genes with transmembrane helices	668	25.21	673	25.49
CRISPR repeats	1		1

**Table 4:** Number of genes associated with general COG functional categories

	SC-57		SC-532		
Code	Value	% age	Value	% age	Description
J	198	8.92	199	9.05	Translation, ribosomal structure and biogenesis
A	82	3.69	81	3.69	RNA processing and modification
K	153	6.89	151	6.87	Transcription
L	102	4.59	100	4.55	Replication, recombination and repair
B	1	0.05	1	0.05	Chromatin structure and dynamics
D	27	1.22	27	1.23	Cell cycle control, Cell division, chromosome partitioning
V	48	2.16	43	1.96	Defense mechanisms
T	67	3.02	67	3.05	Signal transduction mechanisms
M	101	4.55	101	4.6	Cell wall/membrane biogenesis
N	4	0.18	4	0.18	Cell motility
U	14	0.63	14	0.64	Intracellular trafficking and secretion
O	82	3.69	81	3.69	Posttranslational modification, protein turnover, chaperones
C	117	5.27	117	5.32	Energy production and conversion
G	170	7.66	168	7.64	Carbohydrate transport and metabolism
E	206	9.28	205	9.33	Amino acid transport and metabolism
F	88	3.96	86	3.91	Nucleotide transport and metabolism
H	142	6.4	141	6.41	Coenzyme transport and metabolism
I	99	4.46	98	4.46	Lipid transport and metabolism
P	144	6.49	144	6.55	Inorganic ion transport and metabolism
Q	47	2.12	47	2.14	Secondary metabolites biosynthesis, transport and catabolism
R	200	9.01	197	8.96	General function prediction only
S	167	7.52	168	7.64	Function unknown
-	651	24.57	658	24.92	Not in COGs

The total is based on the total number of protein coding genes in the genome

## Insights from the genome sequence

When we compared the metabolic reconstruction of both strains to compare functioning parts we detected 2026 genes associated with a subsystem in both strains (Additional file 1). On the other hand, there are 5 features that were present in strain SC-57 but absent in strain SC-532. One topoisomerase (replication initiation protein), two genes related to sucrose metabolism (one sucrose operon repressor and one sucrose permease), one threonine dehydrogenase, and one L-alanyl-gamma-D-glutamyl-L-diamino acid endopeptidase. These differences were not enough to change the pathway for both strains. In other words, all pathways were the same for both strains (information not shown).

### Extended insights

#### Comparison with 10.1601/nm.5268 (10.1601/strainfinder?urlappend=%3Fid%3DATCC+15305) genome

Based on sequence from all genomes available in the SEED Viewer, both of our strains showed high similarity with other 10.1601/nm.5230 spp. but the closest neighbor with the highest score (523) was 10.1601/nm.5268 (10.1601/strainfinder?urlappend=%3Fid%3DATCC+15305).

Strain SC-57 and SC-532 presented 134 and 131 functioning parts, respectively; which were absent in 10.1601/strainfinder?urlappend=%3Fid%3DATCC+15305 (see Additional file 2). On the other hand, there were 102 and 103 functioning parts that were present in 10.1601/strainfinder?urlappend=%3Fid%3DATCC+15305 but absent in strain SC-57 and SC-532, respectively (see Additional file 3).

##### Biofilm and antibiotic resistance

The biofilm production of each strain was investigated by the Christensen method [25, 27] and both strains were found to be weak biofilm producers. SC-57 and SC-532 presented a biofilm mass with an OD of 0.192 and 0.150, respectively. In the genomes of both isolates, the *icaC* gene for PIA biosynthesis was detected, which may be involved in biofilm production. Antibiotic susceptibility was performed using the broth microdilution method as recommended by the Clinical and Laboratory Standards Institute [33]. The antibiotics tested were erythromycin, trimethoprim, amikacin, vancomycin, linezolid, oxacillin, ciprofloxacin and chloramphenicol (concentrations from 0.125 μg/mL to 512 μg/mL) (Sigma-Aldrich, Toluca, Mexico). Isolate SC-57 was resistant to oxacillin, ciprofloxacin, amikacin, trimethoprim and chloramphenicol. Isolate SC-532 was resistant to oxacillin, amikacin, and trimethoprim. The detection MBEC was performed by the method reported by Ceri, et al. [34]. The MBEC increased significantly (≥2 fold) for amikacin and erythromycin for both isolates and for vancomycin and linezolid for isolate SC-532 (Table 5). Putative genes for resistance to teicoplanin, aminoglycosides, fluoroquinolones, and beta-lactams as well as genes for copper, cobalt, mercury, cadmium, chromium, and arsenic resistance were detected. Both isolates were resistant to the aminoglycoside amikacin, which may be explained by the presence of aminoglycoside adenylyltransferases [35]. Additionally, isolate SC-57 was resistant to ciprofloxacin, which may be associated with mutations in the highly-conserved quinolone resistance determining region of genes that encode DNA gyrase and topoisomerase IV [36].

**Table 5:** Antibiotic resistance of biofilm and planktonic cells of SC-57 and SC-532

		SC-57		SC-532	
Antibiotic	Cells	MIC/MBEC^a^ (μg/mL)	Interpretation^b^	MIC/MBEC^a^ (μg/mL)	Interpretation^b^
Oxacillin	Planktonic	8	R	2	R
	Biofilm	16	R	4	R
Amikacin	Planktonic	*64*	R	*64*	R
	Biofilm	*>256*	R	*>256*	R
Vancomycin	Planktonic	0.25	S	*0.5*	S
	Biofilm	0.5	S	*2*	S
Erythromycin	Planktonic	*0.25*	S	*0.25*	S
	Biofilm	*>1024*	R	*32*	R
Trimethoprim	Planktonic	64	R	16	R
	Biofilm	128	R	32	R
Ciprofloxacin	Planktonic	*8*	R	0.5	S
	Biofilm	*32*	R	0.5	S
Chloramphenicol	Planktonic	32	R	4	S
	Biofilm	32	R	8	S
Linezolid	Planktonic	1	S	*1*	S
	Biofilm	2	S	*8*	R

^a^MIC: minimum inhibitory concentrations (planktonic cells), MBEC Minimum biofilm eradication concentration (biofilm cells). Values in italic indicate a significant difference (increase ≥2 fold) in MICs and MBECs between planktonic and biofilm cells. ^b^ R and S: resistant and susceptible, respectively

Furthermore, genes encoding *bceA*, *bceR* and *bceS* were detected. These genes have been related to bacitracin, mersacidin, and actagardine resistance in 10.1601/nm.10618 [31, 37].

##### Methicillin resistance and SCC *mec* type

Methicillin resistance was determined by the disk diffusion method according to the Clinical and Laboratory Standards Institute [33]. Typing of SCC*mec* elements was performed as previously described by Zhang et al. [38] and Kondo et al. [39]; *ccrAB4* typing was performed using the method described by Oliveira et al. [40] with modifications proposed by Zhang et al. [41]. Both isolates were methicillin resistant and amplified for *mecA*. From both isolates, a new SCC*mec* was detected: *mec* class A, *ccr* type 1. In the genome sequence of both isolates the SCC*mec* had a class A *mec* gene complex composed of methicillin resistance repressor (*mecI*), methicillin resistance regulatory sensor transducer (*mecR1*), penicillin binding protein (PBP2a), and methicillin resistance determinant *mecA* (all within the same contig). Furthermore, cassette chromosome recombinase B (*ccrB*) and cassette chromosome recombinase A (*ccrA*) (both within the same contig), and the insertion sequence IS*431* located in an intergenic region downstream of *mecA* gene, were detected. The nucleotide composition and their location in the genome for both *mecA* and *mecR1* are common to other *Staphylococci* [32]. Interestingly, Zong et al. presented a new SCC*mec* element in 10.1601/nm.11046 and the complete sequence is publicly available (51,384 bp) [42]. This new SCC*mec* possesses a short 99 bp-long sequence between *mecA* and *mecR1* sequences (Additional file 4), which is absent in our strains. This sequence is also absent in the complete sequence of an SCC*mec* genomic island of strain HT20040085 of *Staphylococcus aureus subsp. aureus* (GI:696,158,524) [43]. However, a BLAST search for this short sequence identified hits in 10.1601/nm.11051 (AB353724) with 100% similarity, 10.1601/nm.5250 (AB546266) with 99% similarity, and 10.1601/nm.11043 with 97% similarity.

## Conclusions

10.1601/nm.11046 strains SC-57 and SC-532 were isolated as opportunistic human pathogens. Therefore, their genome sequence will provide insight into the genetic background of virulence and antibiotic resistance of this species. Most genes these strains were involved in metabolism of amino acids and their derivatives, carbohydrates and proteins. Eighty genes were involved in virulence, disease and defense. Both strains showed phenotypic biofilm production and *icaC* gene for PIA biosynthesis was detected in the two genomes. A new SCC*mec* was detected (*mec* class A, *ccr* type 1) for both isolates. We detected evidence of increased antibiotic resistance associated with biofilm production.

## Additional Files

Additional file 1: Comparison of the functioning parts of strain SC-57 and SC-532. (XLSX 85 kb)

Additional file 2: Functioning parts that were present in strain SC-57 and SC-532, but absent in *S. saprophyticus* subsp. *saprophyticus* (ATCC 15305). (PDF 222 kb)

Additional file 3: Functioning parts that were present in *S. saprophyticus* subsp. *saprophyticus* (ATCC 15305) but absent in strain SC-57 and SC-532, respectively (PDF 214 kb)

Additional file 4: Short 99 bp-long sequence between *mecA* and *mecR1* present in the SCC*mec* described by Zong et al. and absent in SC-57 and SC-532 (PDF 81 kb)

## Abbreviations

BLAST: Basic local alignment search tool
COG: Clusters of orthologous groups
CoNS: Coagulase-negative staphylococci
GTR: General Time Reversible
IMG: Integrated Microbial Genomes
MBEC: Minimum Biofilm Eradication Concentration
MIC: Minimum Inhibitory Concentrations
MIGS: Minimum Information about Genome Sequence
OD: Optical density
Pfam: Protein families
PIA: Polysaccharide intercellular adhesin
R: Resistant
RAST: Rapid annotation using subsystem technology
S: Susceptible
SCC*mec*: Staphylococcal Cassette Chromosome *mec*
