# Supplementary material for: Draft genome sequences of two opportunistic pathogenic strains of Staphylococcus cohnii isolated from human patients
Source: Stand Genomic Sci. 2017 Aug 31;12:49. doi: 10.1186/s40793-017-0263-1 (PMC5580220; doi:10.1186/s40793-017-0263-1)
Supplement: Supplementary file 2 — Functioning parts that were present in strain SC-57 and SC-532, but absent in S. saprophyticus subsp. saprophyticus (ATCC 15305). (PDF 222 kb) [file 40793_2017_263_MOESM2_ESM.pdf]

Additional file 2. Genes associated with a subsytem (functioning parts) that were present in our strains (SC-57 and SC-532) but absent in *Staphylococcus saprophyticus* subsp. *saprophyticus* ATCC 15305. We highlighted (bold) the features that are present in only one of our strains.

**Presence in our strains**	**Category**	**Subcategory**	**Subsystem**	**Role**
Both	Aminoacids and Derivatives	Alanine, serine, and glycine	Glycine cleavage system	Sodium/glycine symporter GlyP
Both	Aminoacids and derivatives	Arginine; urea cycle, polyamines	Arginine Biosynthesis -- gjo	Arginine pathway regulatory protein ArgR, repressor of arg regulon
				Predicted arginine uptake transporter
			Arginine deiminase Pathway	Arginine/ornithine antiporter ArcD
				Carbamate kinase
			Arginine and Ornithine Degradation	Ornithine aminotransferase
			Polyamine Metabolism	Transcriptional regulator, MerR family, near polyamine transporter
		Histidine Metabolism	Histidine Biosynthesis	Histidinol-phosphatase [alternative form]
			Histidine Degradation	Histidine transport protein (permease)

		Lysine, threonine, methionine, and cysteine	Cysteine Biosynthesis	Cys regulon transcriptional activator CysB
			Lysine degradation	L-lysine permease
		Proline and 4-hydroxyproline	Proline, 4-hydroxyproline uptake and utilization	Proline/sodium symporter PutP
		no subcategory	Creatine and Creatinine Degradation	Cytosine deaminase
**Present only in SC57282**	Amino Acids and Derivatives	**Lysine, threonine, methionine, and cysteine**	**Threonine degradation**	**Threonine dehydrogenase and related Zn-dependent dehydrogenases**
Both	Protein Metabolism	Protein processing and modification	Inteins	intein-containing
Both	Carbohydrates	Central carbohydrate metabolism	Pyruvate Alanine Serine Interconversions	L-alanine:glyoxylate aminotransferase
		Fermentation	Butanol Biosynthesis	Pyruvate formate-lyase
			Fermentations: Mixed acid	Pyruvate formate-lyase activating enzyme
		Monosaccharides	D-Galacturonate and D-Glucuronate Utilization	2-keto-3-deoxygluconate permease (KDG permease)
				Beta-glucuronidase

		D-mannonate oxidoreductase
		Fructuronate transporter GntP
		Glucuronide transporter UidB
		Mannonate dehydratase
		Predicted D-glucuronide-specific TRAP transporter, substrate-binding component
		Uronate isomerase
	Mannose Metabolism	Activator of the mannose operon (transcriptional antiterminator), BglG family
		Mannose-6-phosphate isomerase
		PTS system, mannose-specific IIA component
		PTS system, mannose-specific IIB component
		PTS system, mannose-specific IIC component
One-carbon Metabolism	Serine-glyoxylate cycle	Serine--pyruvate aminotransferase
Sugar alcohols	Glycerol and Glycerol-3-phosphate Uptake and Utilization	Glycerol-3-phosphate transporter
Aminosugars	Chitin and N-acetylglucosamine utilization	Beta-hexosaminidase

**Present only in SC57282**	**Carbohydrates**	**Di- and oligosaccharides**	**Sucrose utilization**	**Sucrose permease, major facilitator superfamily**
Both	Fatty Acids, Lipids, and Isoprenoids	Fatty acids	Acyl-CoA thioesterase II	TesB-like acyl-CoA thioesterase 2
			Fatty Acid Biosynthesis FASII	Enoyl-[acyl-carrier-protein] reductase [FMN]
		Isoprenoids	Carotenoids	diapolycopene oxidase
				Phytoene desaturase, neurosporene or lycopene producing
				Pro-zeta-carotene desaturase, prolycopene producing
			Isoprenoid Biosynthesis	Isopentenyl-diphosphate delta-isomerase
			Polyprenyl Diphosphate Biosynthesis	diaponeurosporenoate glycosyltransferase
				Dehydrosqualene desaturase;-diapophytoene desaturase)
				Glycosyl-diaponeurosporenoate acyltransferase precursor
		Phospholipids	Glycerolipid and Glycerophospholipid Metabolism in Bacteria	Aldehyde dehydrogenase B

Both	DNA Metabolism	DNA repair	DNA repair, bacterial	DNA repair exonuclease family protein YhaO
			RecFOR pathway	Single-stranded-DNA-specific exonuclease RecJ, Bacteriophage SPBc2-type
			DNA repair, bacterial photolyase	Deoxyribodipyrimidine photolyase
			Metalloendopeptidases (EC 3.4.24.-)	Glycyl-glycine endopeptidase LytM precursor
			Proteolysis in bacteria, ATP-dependent	Putative ATP:guanido phosphotransferase YacI
			Proteolysis in bacteria, ATP-dependent	Transcriptional regulator CtsR
Both	Nucleosides and Nucleotides	no subcategory	Hydantoin metabolism	Beta-ureidopropionase
			Pseudouridine catabolism	Probable pyrimidine nucleoside transport protein associated with pseudouridine catabolism
				Pseudouridine phosphate glycosidase
				Pseudouridine kinase
Both	RNA Metabolism	RNA processing and modification	RNA pseudouridine syntheses	Similar to tRNA pseudouridine synthase C, group TruC1
			tRNA modification Bacteria	tRNA (uridine-5-oxyacetic acid methyl ester) 34 synthase

		Transcription	Transcription initiation, bacterial sigma factors	RNA polymerase sigma factor RpoE
Both	Sulfur Metabolism	Organic sulfur assimilation	L-Cystine Uptake and Metabolism	Bacterial luciferase family protein YtmO, in cluster with L-cystine ABC transporter
Both	Phosphorus Metabolism	no subcategory	Phosphonate metabolism	2-aminoethylphosphonate:pyruvate aminotransferase<br>Phosphonoacetaldehyde hydrolase
Both	Iron acquisition and metabolism	no subcategory	Iron acquisition in Streptococcus	Ferric iron ABC transporter, permease protein
Both	Metabolism of Aromatic Compounds	Metabolism of central aromatic intermediates	Catechol branch of beta-ketoadipate pathway	Beta-ketoadipate enol-lactone hydrolase
Both	Cofactors, Vitamins, Prosthetic Groups, Pigments	Pyridoxine	Pyridoxin (Vitamin B6) Biosynthesis	Pyridoxamine 5'-phosphate oxidase
		Quinone cofactors	Menaquinone and Phylloquinone Biosynthesis	Ubiquinone/menaquinone biosynthesis methyltransferase UbiE

Both	Membrane Transport	ABC transporters	ABC transporter dipeptide (TC 3.A.1.5.2)	Dipeptide transport system permease protein
		Cation transporters	Copper Transport System	Conserved membrane protein in copper uptake, YcnI
		TRAP transporters	TRAP Transporter collection	TRAP-type C4-dicarboxylate transport system, large permease component
				TRAP-type C4-dicarboxylate transport system, small permease component
Both	Phages, Prophages, Transposable elements, Plasmids	Phages, Prophages	Phage capsid proteins	Phage major capsid protein
			Phage introns	HNH homing endonuclease
			Phage packaging machinery	Phage DNA binding protein
				Phage terminase, large subunit
			Phage replication	DNA helicase, phage-associated
				DNA ligase, phage-associated
				DNA primase/helicase, phage-associated
			Phage tail fiber proteins	Phage tail fiber protein
			Phage tail proteins	Phage tail length tape-measure protein

**Present only in SC57282**	**Phages, Prophages, Transposable elements, Plasmids**	**Plasmid related functions**	**Rolling-circle replication**	**Replication initiation protein, topisomerase**
Both	Regulation and Cell signaling	Programmed Cell Death and Toxin-antitoxin Systems	Phd-Doc, YdcE-YdcD toxin-antitoxin (programmed cell death) systems	Death on curing protein, Doc toxin
				Prevent host death protein, Phd antitoxin
			Toxin-antitoxin replicon stabilization systems	HigA protein (antitoxin to HigB)
		Drug resistance or antibiotic biosynthesis related cluster	CBSS-1085.1.peg.1363	Antibiotic biosynthesis monooxygenase
				Transcriptional regulator, ArsR family
		May be related to ADP-phosphoribose and NAD-dependent acetylation	CBSS-216591.1.peg.168	Histone acetyltransferase HPA2 and related acetyltransferases
		Related to Menaquinone-	CBSS-393130.3.peg.129	Unknown conserved protein in B. subtilis

cytochrome C reductase		
		Hypothetical transmembrane protein CDS_ID OB1773
no subcategory	Bacterial Cell Division	Cell division protein FtsX
	CBSS-1313.3.peg.147	FIG006438: hypothetical protein
	CBSS-279010.5.peg.587	hypothetical protein possibly connected to lactam utilization and allophanate hydrolase
	CBSS-349161.4.peg.2427	CBS domain protein, lmo1865 homolog
	CBSS-521098.4.peg.1460	Membrane protein
		Membrane protein
	CBSS-56780.10.peg.1536	Magnesium and cobalt efflux protein CorC
	Disulphide related cluster	Disulfide bond regulator
		Hypothetical protein
		Uncharacterized protein
		Polysulfide binding protein
		Zn-dependent hydroxyacylglutathione hydrolase
	EC699-706	FIG137478: Hypothetical protein YbgI

			LMPTP YfkJ cluster	Hypothetical protein YfkK
		no subcategory	cAMP signaling in bacteria	Adenylate cyclase
				Prophage Clp protease-like protein
Both	Stress Response	Heat shock	Heat shock dnaK gene cluster extended	tRNA-t(6)A37 methylthiotransferase
		Osmotic stress	Choline and Betaine Uptake and Betaine Biosynthesis	Glycine betaine ABC transport system, ATP-binding protein OpuAA
				L-proline glycine betaine binding ABC transporter protein ProX
		Oxidative stress	Glutaredoxins	Glutaredoxin-like protein NrdH, required for reduction of Ribonucleotide reductase class Ib
		no subcategory	Flavohaemoglobin	ABC-type Fe3+-siderophore transport system, permease 2 component
Both	Dormancy and Sporulation	no subcategory	Persister Cells	Cell division inhibitor
Both	Miscellaneous	Plant-Prokaryote DOE project	Iron-sulfur cluster assembly	Ferritin-like protein 2
Both		no subcategory	Muconate lactonizing enzyme family	L-alanyl-gamma-D-glutamyl-L-diamino acid endopeptidase
