# Supplementary material for: Draft genome sequences of two opportunistic pathogenic strains of Staphylococcus cohnii isolated from human patients
Source: Stand Genomic Sci. 2017 Aug 31;12:49. doi: 10.1186/s40793-017-0263-1 (PMC5580220; doi:10.1186/s40793-017-0263-1)
Supplement: Supplementary file 3 — Functioning parts that were present in S. saprophyticus subsp. saprophyticus (ATCC 15305) but absent in strain SC-57 and SC-532, respectively (PDF 214 kb) [file 40793_2017_263_MOESM3_ESM.pdf]

Additional file 3. Genes associated with a subsytem (functioning parts) that were absent in our strains but present in *S. saprophyticus* (D83371, ATCC 15305).

**Absence in our strains**	**Category**	**Subcategory**	**Subsystem**	**Role**
Both	Carbohydrates	Central carbohydrate metabolism	Pyruvate metabolism I: anaplerotic reactions, PEP	Pyruvate carboxyl transferase
		Di- and oligosaccharides	Sucrose utilization	PTS system, sucrose-specific IIB component
				PTS system, sucrose-specific IIC component
				Sucrose-6-phosphate hydrolase
		Fermentation	Acetoin, butanediol metabolism	Acetolactate synthase, catabolic
			Acetyl-CoA fermentation to Butyrate	Phosphate butyryltransferase
		Monosaccharides	D-gluconate and ketogluconates metabolism	Gluconate transporter family protein
			Xylose utilization	D-xylose transport ATP-binding protein XylG
		One-carbon Metabolism	Formaldehyde assimilation: Ribulose monophosphate pathway	D-arabino-3-hexulose 6-phosphate formaldehyde lyase

				Transcriptional regulator HxlR, formaldehyde assimilation
		Sugar alcohols	Glycerol and Glycerol-3-phosphate Uptake and Utilization	Glycerol-3-phosphate responsive antiterminator (mRNA-binding)
		Organic acids	Alpha-acetolactate operon	Transcriptional regulator of alpha-acetolactate operon AlsR
**Absent only in SC532283**	**Carbohydrates**	**Di- and oligosaccharides**	**Sucrose utilization**	**Sucrose operon repressor ScrR, LacI family**
Both	Fatty Acids, Lipids, and Isoprenoids	Fatty acids	Fatty acid metabolism cluster	3-oxoacid CoA-transferase
		Isoprenoids	Isoprenoid Biosynthesis	2-C-methyl-D-erythritol 4-phosphate cytidylyltransferase
Both	Amino Acids and Derivatives	Alanine, serine, and glycine	Alanine biosynthesis	Putative cysteine desulfurase, associated with tRNA 4-thiouridine synthase
		Arginine; urea cycle, polyamines	Arginine Biosynthesis -- gjo	Acetylornithine aminotransferase 2
			Arginine and Ornithine Degradation	Delta-1-pyrroline-5-carboxylate dehydrogenase
		Aromatic amino acids and derivatives	Chorismate: Intermediate for	Isochorismatase

			synthesis of Tryptophan, PAPA antibiotics, PABA, 3-hydroxyanthranilate and more.	
		Branched-chain amino acids	Isoleucine degradation	Acyl-CoA dehydrogenase, short-chain specific
		Lysine, threonine, methionine, and cysteine	Cysteine Biosynthesis	Cystathionine beta-synthase
				Cystathionine gamma-lyase
Both	RNA Metabolism	RNA processing and modification	ATP-dependent RNA helicases, bacterial	ATP-dependent RNA helicase YqfR
				Cold-shock DEAD-box protein A
			Queuosine-Archaeosine Biosynthesis	NADPH-dependent 7-cyano-7-deazaguanine reductase
			RNA methylation	23S rRNA (Uracil-5-) -methyltransferase RumA
			tRNA modification Bacteria	tRNA (cytidine(34)-2'-O)-methyltransferase
Both	DNA Metabolism	DNA recombination	RuvABC plus a hypothetical	FIG000859: hypothetical protein YebC
		DNA repair	DNA repair, bacterial RecFOR pathway	ATP-dependent DNA helicase RecS (RecQ family)

		DNA replication	DNA replication strays	Probabl; exonuclease Bsu YpcP
		DNA uptake, competence	Late competence	Late competence protein ComGE, FIG018915<br>Late competence protein ComGF, access of DNA to ComEA, FIG017774<br>Capsular polysaccharide synthesis enzyme Cap8D<br>Capsular polysaccharide synthesis enzyme Cap8M<br>Capsular polysaccharide synthesis enzyme Cap8N
		Gram-Positive cell wall components	Teichoic and lipoteichoic acids biosynthesis	Putative polyribitolphosphotransferase
			Teichuronic acid biosynthesis	Putative N-acetylgalactosaminyl-diphosphoundecaprenol glucuronosyltransferase
		no subcategory	Peptidoglycan Biosynthesis	D-alanine--D-alanine ligase A
			Recycling of Peptidoglycan Amino Sugars	N-acetylmuramic acid 6-phosphate etherase
Both	Nucleosides and Nucleotides	Purines	De Novo Purine Biosynthesis	YabJ, a purine regulatory protein and member of the

				highly conserved YjgF family
Both	Protein Metabolism	Protein biosynthesis	Programmed frameshift	Peptide chain release factor 2 unshifted fragment
		Protein degradation	Protein degradation	Deblocking aminopeptidase
Both	Phosphorus Metabolism	no subcategory	Alkylphosphonate utilization	Alkylphosphonate utilization operon protein PhnA
Both	Cofactors, Vitamins, Prosthetic Groups, Pigments	Folate and pterines	5-FCL-like protein	Butyryl-CoA dehydrogenase
				Phosphomethylpyrimidine kinase
				Thiaminase II
		Riboflavin, FMN, FAD	Flavodoxin	Flavodoxin 2
				NAD(P)H oxidoreductase YRKL (EC 1.6.99.-)
			Riboflavin, FMN and FAD metabolism in plants	Molybdopterin binding motif, CinA N-terminal domain
Both	Clustering-based subsystems	Clustering-based subsystems	CBSS-262719.3.peg.410	Replicative DNA helicase
		DNA metabolism	Rad50-Mre11 DNA repair cluster	DNA double-strand break repair protein Mre11
		Nucleotidyl-phosphate metabolic cluster	CBSS-222523.1.peg.1311	Adenylate cyclase

		no subcategory		Inner membrane protein YihY, formerly thought to be RNase BN
		no subcategory	CBSS-196620.1.peg.2477	Copper ion binding protein
			Conserved gene cluster associated with Met-tRNA formyltransferase	16S rRNA (cytosine(967)-C(5))-methyltransferase
			Possible Ammonia conversion cluster	FIG025881: hypothetical protein in Ammonia conversion cluster
Both	Dormancy and Sporulation	no subcategory	Persister Cells	Cell division inhibitor Slr1223 (YfcH in EC), contains epimerase/dehydratase and DUF1731 domains
Both	Membrane Transport	ABC transporters	ABC transporter alkylphosphonate (TC 3.A.1.9.1)	Phosphonate ABC transporter ATP-binding protein
				Phosphonate ABC transporter permease protein phnE1
				Phosphonate ABC transporter permease protein phnE2
				Phosphonate ABC transporter phosphate-

				binding periplasmic component
			ABC transporter dipeptide (TC 3.A.1.5.2)	Dipeptide transport ATP-binding protein DppD
				Oligopeptide transport ATP-binding protein OppD
		Uni- Sym- and Antiporters	Multi-subunit cation antiporter	Na(+) H(+) antiporter subunit A
				Na(+) H(+) antiporter subunit D
				Na(+) H(+) antiporter subunit E
				Na(+) H(+) antiporter subunit F
			Proton-dependent Peptide Transporters	Di/tripeptide permease YjdL
Both	Regulation and Cell signaling	Programmed Cell Death and Toxin-antitoxin Systems	MazEF toxin-antitoxing (programmed cell death) system	Programmed cell death antitoxin YdcD
		no subcategory	Cell envelope-associated LytR-CpsA-Psr transcriptional attenuators	Manganese-dependent protein-tyrosine phosphatase
				Tyrosine-protein kinase EpsD

				Tyrosine-protein kinase transmembrane modulator EpsC
			HPr catabolite repression system	Phosphotransferase system, phosphocarrier protein HPr
			Staphylococcal accessory gene regulator system	Accessory gene regulator C (sensor histidine kinase)
				Major cold shock protein CspA
				Signal transduction protein TRAP (Target of RNAIII-activating protein)
				Staphylococcal respiratory response protein SrrB
Both	Respiration	ATP synthases	F0F1-type ATP synthase	ATP synthase F0 sector subunit a
				ATP synthase F0 sector subunit b
				ATP synthase F0 sector subunit c
				FIG048548: ATP synthase protein I2
		no subcategory	Formate hydrogenase	NAD-dependent formate dehydrogenase
Both	Secondary Metabolism	Plant Hormones	Auxin biosynthesis	Indole-3-pyruvate decarboxylase

Both	Stress Response	Cold shock	Cold shock, CspA family of proteins	Cold shock protein CspG
		Osmotic stress	Choline and Betaine Uptake and Betaine Biosynthesis	L-proline glycine betaine ABC transport system permease protein ProW
			Osmoregulation	Aquaporin Z
		Periplasmic Stress	Periplasmic Stress Response	Intramembrane protease RasP/YluC, implicated in cell division based on FtsL cleavage
		no subcategory	SigmaB stress responce regulation	Anti-sigma B factor antagonist RsbV
				Anti-sigma F factor antagonist (spoIIAA-2)
Both	Virulence, Disease and Defense	Resistance to antibiotics and toxic compounds	Aminoglycoside adenylyltransferases	Aminoglycoside N6'-acetyltransferase
			Arsenic resistance	Arsenical pump-driving ATPase
				Arsenical resistance operon trans-acting repressor ArsD
			Copper homeostasis: copper tolerance	Cytoplasmic copper homeostasis protein CutC
			Fosfomycin resistance	Fosfomycin resistance protein FosB
			Methicillin resistance in Staphylococci	HmrB protein involved in methicillin resistance

				UDP-N-acetylmuramoylalanyl-D-glutamate--2,6-diaminopimelate ligase
			Multidrug Resistance Efflux Pumps	Multi antimicrobial extrusion protein (Na(+)/drug antiporter), MATE family of MDR efflux pumps
Both	Miscellaneous	Plant-Prokaryote DOE project	Conserved gene cluster possibly involved in RNA metabolism	TrmH family tRNA/rRNA methyltransferase YacO
