# Supplementary material for: Draft genome sequences of two opportunistic pathogenic strains of Staphylococcus cohnii isolated from human patients
Source: Stand Genomic Sci. 2017 Aug 31;12:49. doi: 10.1186/s40793-017-0263-1 (PMC5580220; doi:10.1186/s40793-017-0263-1)
Supplement: Supplementary file 4 — Short 99 bp-long sequence between mecA and mecR1 present in the SCCmec described by Zong et al. and absent in SC-57 and SC-532 (PDF 81 kb) [file 40793_2017_263_MOESM4_ESM.pdf]

This new SCC*mec* element also possesses the MecA and MecR1 sequences but also possess in between a short 99 bp-long sequence (CTGCTACACCTCCATATCACAAAAATTATAACATTATTTTGACATAAACACTACATTTGTAATATACTACAAATGTAGTCCTATATAAGGAGGATATTG), which is absent in our strains.
